# Performing newborn life support in advance of neonatal advanced life support course—back to basics?

**DOI:** 10.1007/s00431-020-03917-9

**Published:** 2021-01-13

**Authors:** Tim Hundscheid, Jos Bruinenberg, Jeroen Dudink, Rogier de Jonge, Marije Hogeveen

**Affiliations:** 1 Division of Neonatology, Department of Paediatrics, Amalia Children’s Hospital, Radboud Institute for Health Sciences, Radboud University Medical Centre, Geert Grooteplein Zuid 10, 6525 GA Nijmegen, The Netherlands; 2 Department of Paediatrics, ETZ Hospital, Hilvarenbeekseweg 60, 5022 GC Tilburg, The Netherlands; 3 Division of Neonatology, Department of Paediatrics, Wilhelmina Children’s Hospital, University Medical Centre Utrecht, Lundlaan 6, 3584 EA Utrecht, The Netherlands; 4 Pediatric Intensive Care Unit, Departments of Pediatrics and Pediatric Surgery, Erasmus MC - Sophia Children’s Hospital, Postbus 2060, 3000 CB Rotterdam, The Netherlands

**Keywords:** Newborn life support, Airway management, Retention of skills, Simulation

## Abstract

In this retrospective analysis, the Newborn Life Support (NLS) test scenario performance of participants of the Dutch Neonatal Advanced Life Support (NALS) course was assessed. Characteristics of participants and total amount of failures were collected. Failures were subdivided in (1) errors of omission; (2) errors of commission; and (3) unspecified if data was missing. Pearson’s chi-squared test was used to assess differences between participant groups. In total, 23 out of 86 participants (27%) failed their NLS test scenario. Life support course instructors in general (20/21) passed their test scenario more often compared to other participants (43/65) (*p* = 0.008). In total 110 fail items were recorded; the most common errors being not assessing heart rate (error of omission) (*n* = 47) and inadequate performance of airway management (error of commission) (*n* = 24).

*Conclusion*: A substantial part of NALS participants failed their NLS test scenario. Errors of omission could be reduced by the availability of a checklist/NLS algorithm. Life support course instructors possibly make less errors of commission due to retention of skills by teaching these skills at least twice a year. Therefore, our study suggests that neonatal basic life support skills should be retained by local assurance of training programmes.

**Table:** 

**What is Known:** *• Retention of skills after life support courses decreases after three months.* *• Adherence to newborn life support guidelines is suboptimal*.	
**What is New:** *• NLS performance is suboptimal in participants for advanced neonatal life support.* *• Most common failures are not assessing heart rate and inadequate airway management.*

**Supplementary Information:**

The online version contains supplementary material available at 10.1007/s00431-020-03917-9.

## Introduction

The 1-day Newborn Life Support® (NLS) course, developed by the European Resuscitation Council, has been available in The Netherlands since 2003 for all health care professionals involved in the delivery of newborns. While sufficient for most participants, paediatricians expressed the need for more complex scenarios, scenarios beyond the delivery room and more advanced airway skills. Furthermore, there has been growing interest in crew resource management (CRM) skills in the assessment and treatment of critically ill patients [1]. While scenarios in the NLS course consist of the format of one physician working with a non-obstructive nurse, in real life CRM skills are essential in (neonatal) emergency care. To fulfill this need, the Dutch Foundation for the Emergency Medical Care of Children (*Stichting Spoedeisende Hulp bij Kinderen—*SHK) recently developed the 2-day Neonatal Advanced Life Support® (NALS) course.

**Fig. 1:**
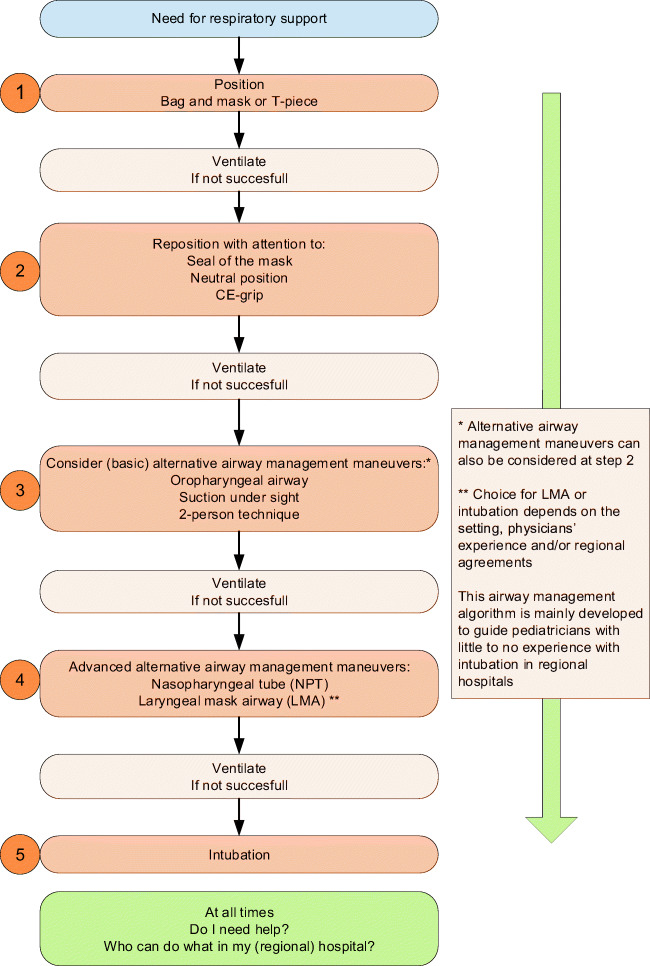
Airway management algorithm as used in Newborn Advanced Life Support course

This course offers additional theoretical education and skill training regarding airway management, more complex simulations beyond the context of the delivery room, and CRM skills, as compared to the NLS course. It focuses on early recognition of neonatal compromise which warrants structured analysis and treatment. This course is explicitly not meant for teaching neonatal intubation skills, since this is not feasible in a 2-day course [2]. Alternatively, it merely focuses on basic and alternative airway manoeuvres (Fig. 1).

As adequate NLS performance is the starting point of the NALS course, this was assumed to be sufficient and formally tested by a pre-announced pre-course practical test on the first day of the NALS course. Being NLS certified was not a prerequisite, since retention of skills and knowledge after life support courses, both in general and specifically NLS, deteriorates as early as 3 months after the life support course [3, 4]. This situation provides a unique opportunity to assess practical skills on a manikin in a group of neonatal and paediatric physicians.

## Methods

### Design/study group

This study is a retrospective analysis of scoring forms of the pre-course practical test scenario of participants on the first six NALS courses. Our institutional review board stated no ethical approval was needed according to the Dutch Medical Research Involving Human Subjects Act. All candidates from the NALS received a practical pre-course test. The participants were informed several weeks prior to the NALS course about the practical pre-course test in which no guideline or flowchart would be available. Assessments were made by two experienced senior NLS instructors simultaneously. In total, sixteen different instructors examined the participants during the first six NALS courses. One instructor performs the assessment, while the other is the observing instructor. Only after mutual agreement the decision of a pass or fail of the practical pre-course test was made. Participants who failed this test were offered a ‘booster session’ on airway management and/or NLS algorithm during the NALS course and were retested on the practical pre-course test at the end of the NALS course. Participants who also failed their retest were not NALS certified at the end of the 2-day course.

### Inclusion criteria

All completed pre-course tests were included for analysis.

### Exclusion criteria

Incomplete pre-course tests were excluded.

### Description of the practical pre-course test

The pre-course test assesses NLS scenario performance and practical airway management skills on a newborn manikin. Initial bag and mask ventilation and regular checks of vital functions are assessed. If ventilation fails, alternative airway management techniques (two-person technique, suction under direct vision and insertion of an oropharyngeal airway) should be employed and are assessed on their efficacy. The pre-course test is based on an alignment of the European Resuscitation Council 2015 guidelines on neonatal resuscitation and can be accessed as supplementary material [5].

### Definition of potential errors

Errors made during the pre-course test were divided in four subgroups, namely (1) failure in airway management; (2) failure in ventilatory support; (3) failure to assess heart rate; and (4) failure in thermal management. To gain insight in which kind of errors were made, we subdivided errors in (1) errors of omission (an error which occurs as a result of an action or assessment not taken); (2) errors of commission (an error which occurs as a result of not timely, or technically incorrect, performing an indicated action); and (3) unclassified if data was missing on the scoring forms [6].

### Statistical analysis

Baseline characteristics of participants were collected for NLS certification status, SHK instructor status (NLS or other life support course), (sub)specialty and type of hospital they currently worked. For each participant, total amount of recorded fail items were collected and assessed for type of error. Pearson’s chi-squared test was used to assess differences between participant groups. All statistical data analyses were performed using SPSS version 25.0 (IBM Corp. 2017).

## Results

During the first six NALS courses, 86 participants attended the course. Baseline characteristics could be recruited of 85 participants (99%). Out of 86 participants, 23 (27%) failed their NLS test scenario (Table 1). Participants who are SHK life support course instructors (20/21) passed their pre-course test more often (*p* = 0.008) compared to other participants (43/65).

**Table 1:** Characteristics of NALS course participants stratified by NLS test results

		Pass (*n* = 63)	Fail (*n* = 23)
NLS certified (*n* = 59)	Yes; *n* (%)	46 (73)	13 (56.5)
	Years since NLS course; median [IQR]	5 [3;8]	6 [2;9.5]
SHK instructor (*n* = 21)	NLS*	14	0
	Other life support course(s)*	6	1
(Sub)specialty (*n* = 85)	Neonatologist/fellow	9 (14.5)	4 (17.4)
	Paediatrician	22 (35.5)	8 (34.8)
	Paediatric resident (not) in training	18 (29.0)	8 (34.8)
	Physician assistant/nursing specialist	10 (16.1)	2 (8.7)
	Other	3 (4.8)	1 (4.3)
Hospital (*n* = 85)	NICU	13 (21.0)	5 (21.7)
	Academic hospital–not NICU	14 (22.6)	7 (30.4)
	Teaching hospital	19 (30.6)	4 (17.4)
	General hospital	15 (24.2)	7 (30.4)
	Abroad	1 (1.6)	0

*IQR*, interquartile range; *NICU*, neonatal intensive care unit; *NLS*, Newborn Life Support; *SHK*, Dutch Foundation for the Emergency Medical Care of Children

*p < 0.05

In total 110 fail items were recorded, of which 14 were made by participants who passed (*n* = 63) their pre-course test and 96 by participants who failed (*n* = 23) it. Most commonly the error of omission not assessing heart rate (*n* = 47) and the error of commission inadequate performance of airway management (*n* = 24) were made (Supplementary Table 1).

Of the 23 participants who failed their first pre-course test, 21 (91%) passed their retest while 2 (9%) failed and were therefore not NALS certified at the end of the 2-day course.

## Discussion

The ultimate goal of neonatal resuscitation is to re-establish adequate respiration and cardiac output to prevent the morbidity and mortality associated with hypoxic-ischaemic tissue [5]. Therefore, airway management skills are of utmost importance and are the key content of the NLS course. Due to significant less indications for intubation and invasive ventilation in newborns, the exposure to neonatal intubation has dropped drastically. The lack of intubation skills of paediatricians resulting from the decreased exposure during paediatric resident training, and in general practice therefore warrants adequate alternative airway management skills [2].

It has been shown that retention of theoretical knowledge is more sustainable than practical skills [7]. Therefore, we focused on pre-course practical test performance. Although there is not a direct linear correlation between demonstrating practical skills on a manikin and performance in a real-life situation, it is generally accepted that there is a certain amount of transfer of skills in real life [8].

A substantial part of NALS participants (27%) failed their pre-course test. Most errors made were errors of omission, especially not assessing heart rate, which is in line with a previous study [3]. Inadequate performance of airway management skills was the most common error of commission. SHK life support course instructors might make less errors of commission due to retention of skills by teaching, and thereby also practicing, them at least twice a year [3].

Although the pre-course test was pre-announced, the overall performance was in line with a recent publication on paediatricians ad hoc [9], and paediatric residents induction NLS performance [4].

We found no difference in pre-course test performance in relation to time since last NLS certification. This supports SHK’s statement that NLS certification is not a prerequisite for NALS course participation, since retention of skills is much shorter than the 5-year period between (re)certification [3].

One limitation of our study is the retrospective analysis of pre-course test scoring forms. Twenty-seven percent of recorded errors could not be specified. All instructors taking the exams were senior NLS instructors. Since agreement between both NLS instructors is needed, inter-observer differences are minimized. This is important, since previous data shows that there might be significant inter-observer differences in life support course assessment [10].

Secondly, although baseline characteristics could be recruited for most participants, they were limited. Data on participants’ exposure to clinical NLS scenarios and/or frequency of in-hospital NLS training was not known. As training frequency greatly varies between hospitals [11], this might be a major contributor to retention of skills and thereby better pre-course test performance. Previous studies have shown a significant correlation between both exposure to neonatal resuscitation and frequency of neonatal resuscitation training and test performance [3].

Apart from the available N(A)LS course, a local programme to assure acquisition and retention of skills and availability of checklists could be useful. Local training might improve clinical practice [12]. Such a local programme may consist of high-frequency but low-dose skills training. Since life support course skills deteriorate as early as 3 months after the course [3], we suggest on-the-job training sessions at least every 3 to 6 months, depending on the clinical exposure to neonatal resuscitation.

A substantial part of NALS participants failed their pre-announced pre-course NLS test, mainly due to not assessing heart rate (error of omission) and inadequate airway management (error of commission). Life support course instructors perform better, supposedly due to retention of skills.

To improve NLS performance, apart from the available N(A)LS course(s), local availability of checklists and NLS algorithm and assurance of retention of basic, but potentially lifesaving, skills at least twice a year is warranted to ensure proper delivery of airway management techniques in a compromised newborn as demonstrated on a manikin.

## Supplementary Information

ESM 1 (DOCX 14 kb)

ESM 2 (DOCX 25 kb)

## Data Availability

Data set will be shared upon reasonable request.

## Abbreviations

CRM: Crew resource management
NALS: Newborn Advanced Life Support®
NLS: Newborn Life Support®
SHK: Dutch Foundation for the Emergency Medical Care of Children (*Stichting Spoedeisende Hulp bij Kinderen)*
