# Supplementary material for: Performing newborn life support in advance of neonatal advanced life support course—back to basics?
Source: Eur J Pediatr. 2021 Jan 13;180(5):1647–51. doi: 10.1007/s00431-020-03917-9 (PMC8032610; doi:10.1007/s00431-020-03917-9)
Supplement: Supplementary file 1 — (DOCX 14 kb) [file 431_2020_3917_MOESM1_ESM.docx]

Supplementary Table 1 – Fail items and type of error recorded during first NLS test scenario

|  | | Pass  (n=63 participants) | Fail  (n=23 participants) |
| --- | --- | --- | --- |
| Total fail items  (n=110) | Errors of omission | 7 | 53 |
|  | Errors of commission | 3 | 32 |
|  | Unspecified | 4 | 11 |
| Failure to assess heart rate  (n=50) | Errors of omission | 6 | 41 |
|  | Errors of commission | 0 | 0 |
|  | Unspecified | 1 | 2 |
| Failure in airway management  (n=40) | Errors of omission | 1 | 8 |
|  | Errors of commission | 2 | 22 |
|  | Unspecified | 1 | 6 |
| Failure in ventilatory support  (n=13) | Errors of omission | 0 | 3 |
|  | Errors of commission | 0 | 6 |
|  | Unspecified | 2 | 2 |
| Failure in thermal management  (n=7) | Errors of omission | 0 | 1 |
|  | Errors of commission | 1 | 4 |
|  | Unspecified | 0 | 1 |
