# Supplementary material for: Performing newborn life support in advance of neonatal advanced life support course—back to basics?
Source: Eur J Pediatr. 2021 Jan 13;180(5):1647–51. doi: 10.1007/s00431-020-03917-9 (PMC8032610; doi:10.1007/s00431-020-03917-9)
Supplement: Supplementary file 2 — (DOCX 25 kb) [file 431_2020_3917_MOESM2_ESM.docx]

**Supplement 1 –** Newborn Advanced Life Support practical pre-course test, adapted from the European Resuscitation Council (5)

| ***With this test scenario we would like to assess your newborn life support and airway management skills.***  ***In this test scenario a term newborn is born blue and hypotonic. Please show me how you assess and resuscitate the baby.*** | | | |
| --- | --- | --- | --- |
|  |  |  |  |
| 1. | Start clock, put on heater, shout for help |  |  |
| 2. | Dry the baby, remove wet towels and cover the baby with dry towels |  |  |
| 3. | Assess breathing and heartrate |  |  |
|  | ***“The baby is hypotonic, blue, has no spontaneous breathing and has a low heartrate”*** |  |  |
| 4. | Airway management, with head in neutral position and jaw-thrust |  |  |
| 5. | Picks right mask and size for ventilation and places it correct (C+E grip) |  |  |
| 6. | Gives 5 inflation breaths for 2-3 seconds/inflation |  |  |
| 7. | Check effect: has the heartrate increased? (If not, have chest movements been seen?) |  |  |
|  | ***“The chest did not move and the heartrate remains low, what would be your next step?”*** |  |  |
| 8. | Reposition: check neutral position, jaw-thrust and mask and repeat insufflation breaths |  |  |
| 9. | Check effect: has the heartrate increased? (If not, have chest movements been seen?) |  |  |
| 10. | Consider pulse oximetry measurement on the right arm |  |  |
|  | ***“There has not been a change, the chest did not move and the heartrate remains low, what would be your next step?”*** |  |  |
|  | The participant should choose one of the following maneuvers: |  |  |
| 11. | 2 person technique with jaw thrust and repeat inflation breaths; |  |  |
| 12. | and/or inspection and suction of the oropharynx under sight (i.e. laryngoscope or tongue depressor) and repeat inflation breaths; |  |  |
| 13. | and/or oropharyngeal airway and repeat inflation breaths; |  |  |
|  | ***Whatever method the participants has chosen, this time it is effective if performed correct.***  ***The other maneuvers which have not been used during the simulation will be assessed at the end of the test scenario.*** |  |  |
| 14. | Check effect: has the heartrate increased? (If not, have chest movements been seen?) |  |  |
|  | **“The heartrate remains low, but during the inflation breaths the chest did move”** |  |  |
| 15. | Confirm chest movements with 30 seconds ventilation |  |  |
|  | **“The heartrate remains low, but during the inflation breaths the chest did move”** |  |  |
| 16. | Indicate the need for chest compressions |  |  |
| 17. | Start with chest compressions and continue ventilation (3:1) |  |  |
| 18. | Consider increasing the FiO_2_ |  |  |
|  | ***After 30 seconds of chest compressions and ventilation the scenario is stopped.***  ***Ask the participant if the chosen airway maneuver would not have been successful which other maneuvers could have been used. Assess technique.*** |  |  |
|  |  |  |  |

| PASS |  | FAIL |  |
| --- | --- | --- | --- |
| ***All airway maneuvers need to be performed on the manikin in a correct way in order to PASS.***  ***Orange boxes are no formal PASS/FAIL items.***  ***In case another red boxed PASS/FAIL item is not scored, it is at the discretion of both instructors to decide after mutual agreement whether to conclude a practical pre-course test PASS or FAIL.***  ***If a participant has failed the first test, the same test is assessed again at the end of the Newborn Advanced Life Support course.*** | | | |
